# Editorial: The neurobiology of suicide: the ‘suicidal brain'

**DOI:** 10.3389/fpsyt.2023.1215902

**Published:** 2023-06-09

**Authors:** Cicek Hocaoglu

**Affiliations:** Department of Psychiatry, Recep Tayyip Erdogan University, Rize, Türkiye

**Keywords:** suicide, neurobiology, biomarker, suicidal behavior, suicidal brain

Suicide is a growing public health problem (1, 2). It can be seen in a wide range of people, from mentally healthy individuals who react to challenging living conditions to patients with severe mental disorders (3–5). Suicide has complex biological, social, and psychological risk factors and a multidimensional clinical presentation (6, 7). Recent studies have revealed the complexity underlying the neurobiological mechanisms of suicide (8–12). This Research Topic includes 10 studies examining the neurobiological causes of suicidal behavior. In the first article, Jiang et al. investigated the relationship between plasma inflammatory cytokine levels and changes in brain white matter (WM) integrity in patients with bipolar disorder who attempted suicide. Although no significant relationship was found between plasma inflammatory cytokines and WM integrity in the study, the increase in IL-6 levels was remarkable. The results of this study may provide a scientific basis for understanding abnormal immunological and neuroimaging changes in the possible mechanisms of suicidal behavior in patients with bipolar disorder (Jiang et al.). Secondly, Genis-Mendoza, Dionisio-García, et al. examined increased cortisol levels and number of suicide attempts and its relationship with depression. In this study, plasma cortisol levels were found to be high in individuals with depression and two or more suicide attempts. The authors stated that cortisol levels can be taken into account in people who attempt suicide and can be evaluated as a marker in the prevention of this global problem (Genis-Mendoza, Dionisio-García, et al.). In the third article, Kim et al. examined impaired oxygenation of the prefrontal cortex by functional near-infrared spectroscopy (fNIRS) during a verbal fluency task in young adults with major depressive disorder and suicidal behavior. In this study, it is noteworthy that a significantly impaired prefrontal oxygenation was obtained, especially in the right ventrolateral prefrontal cortex (VLPFC) in major depressive disorder (MDD) patients with suicidal tendencies. Impaired prefrontal oxygenation during cognitive execution may serve as a diagnostic biomarker for suicidality in young adult patients with MDD (Kim et al.). In a systematic review, Genis-Mendoza, Hernández-Díaz, et al. examined the relationship between TPH1 polymorphisms and the risk of suicidal behavior. The authors reported that the A218C polymorphism of the TPH1 gene may be a possible risk factor for suicide as a result of the study designed as an updated meta-analysis of 18.398 individuals (Genis-Mendoza, Hernández-Díaz, et al.). In the fifth article, Li X. et al. examined changes in whole-brain gray matter volumes (GMVs) before and after electroconvulsive therapy (ECT) in adolescents with MDD and suicidal ideation. The authors reported increased GMV in the right superior frontal gyrus and right superior temporal gyrus after ECT. They also reported that frontal-temporal-precuneus structure changes may be a potential cause of depressive and suicidal symptoms in adolescents (Li X. et al.). In the sixth study, Li J. et al. examined the associations between anxiety, depression, and risk of suicidal behavior in Chinese medical school students. The authors emphasized the importance of screening for anxiety and depressive symptoms when assessing the risk of suicidal behavior and reducing anxiety in addition to depressive symptoms in treatment (Li J. et al.). In this review, Dobbertin et al. evaluated the current results of MRI studies examining the neuroimaging changes of the suicidal brain and its relevance to practice. The authors reported that there were morphological changes in brain neuroimaging studies, especially in the frontolimbic network, and there was evidence pointing to deterioration in cognitive functions (Dobbertin et al.). In the eighth article, Koseki et al. examined a pharmacovigilance approach to assess the occurrence of suicidal events induced by antiepileptic drugs using the Japanese adverse drug event report database. The increased suicidality rate after antiepileptic drug (AED) therapy is controversial. The authors suggested that young women using more than one antiepileptic drug especially should be warned about suicidal behavior in the first months of treatment (Koseki et al.). In the ninth article, Tsiouris and Flory compared the downregulation of cyclic adenosine monophosphate levels in leukocytes of hibernating black bears with the findings of cyclic adenosine monophosphate reported in major depressive disorder. It has been reported in previous studies that cyclic adenosine monophosphate (cAMP) levels in lymphoblasts and leukocytes of patients with major depressive disorder (MDD) are downregulated compared to controls. Similarities have been noted between the neurobiological changes associated with MDD in humans and many conditions associated with mammalian hibernation. The authors noted that they resemble neurobiological findings associated with hypometabolism (metabolic depression) observed during mammalian hibernation and reported during MDD (Tsiouris and Flory). In the last study, da Silva Schmidt et al. sought an answer to the question of whether glutathione could be a biomarker for suicide risk in women 18 months after birth. In this study, it was concluded that there is a relationship between low glutathione levels in the postpartum period and suicide risk. The authors noted that glutathione may be a potential biomarker or etiological factor in women at moderate to high risk of suicide (da Silva Schmidt et al.).

We would like to thank Frontiers for their interest and support in the planning of our Research Topic. We would also like to thank all the authors and referees who contributed to the preparation of the Research Topic.

The pandemic that affected our world during our Research Topic changed our lives. Then, a short time ago, an earthquake disaster that took place in our country, Turkey, increased our suffering even more. I respectfully commemorate all the souls who passed away in both life events and the significant stress created because of them. Continuing to work, produce, and contribute to science without losing hope and increasing solidarity despite all difficulties should be among our top priorities.

In conclusion, I think that the Research Topic provides an assessment of neurobiological risk factors for suicidal behavior that will play an important role in the prevention of suicide, as well as in the regulation of treatment algorithms and in the follow-up of treatment.

## Author contributions

The author confirms being the sole contributor of this work and has approved it for publication.
